# Application of a High-Throughput Targeted Sequence AmpliSeq Procedure to Assess the Presence and Variants of Virulence Genes in *Salmonella*

**DOI:** 10.3390/microorganisms10020369

**Published:** 2022-02-05

**Authors:** Ruimin Gao, Hongsheng Huang, Jérémie Hamel, Roger C. Levesque, Lawrence D. Goodridge, Dele Ogunremi

**Affiliations:** 1 Ottawa Laboratory Fallowfield, Canadian Food Inspection Agency, Ottawa, ON K2J 4S1, Canada; hongsheng.huang@inspection.gc.ca; 2 Department of Food Science and Agricultural Chemistry, McGill University, Ste Anne de Bellevue, QC H9X 3V9, Canada; 3 Merinov, 96 Montée Sandy Beach, Gaspé, Quebec, QC G4X 2V6, Canada; jeremie.hamel@hotmail.com; 4 Institut de Biologie Intégrative et des Systèmes, Université Laval, Quebec, QC G1V 0A6, Canada; rclevesq@ibis.ulaval.ca; 5 Department of Food Science, University of Guelph, Guelph, ON N1G 2W1, Canada; goodridl@uoguelph.ca

**Keywords:** AmpliSeq, virulence genes, presence/absence, serovar, variant, pathogenicity, food safety, *Salmonella*

## Abstract

We have developed a targeted, amplicon-based next-generation sequencing method to detect and analyze 227 virulence genes (VG) of *Salmonella* (AmpliSeq_Salm_227VG_) for assessing the pathogenicity potential of *Salmonella*. The procedure was developed using 80 reference genomes representing 75 epidemiologically-relevant serovars associated with human salmonellosis. We applied the AmpliSeq_Salm_227VG_ assay to (a) 35 previously characterized field strains of *Salmonella* consisting of serovars commonly incriminated in foodborne illnesses and (b) 34 *Salmonella* strains with undisclosed serological or virulence attributes, and were able to divide *Salmonella* VGs into two groups: core VGs and variable VGs. The commonest serovars causing foodborne illnesses such as Enteritidis, Typhimurium, Heidelberg and Newport had a high number of VGs (217–227). In contrast, serovars of subspecies not commonly associated with human illnesses, such as houtenae, arizonae and salame, tended to have fewer VGs (177–195). Variable VGs were not only infrequent but, when present, displayed considerable sequence variation: *safC*, *sseL*, *sseD*, *sseE*, *ssaK* and *stdB* showed the highest variation and were linked to strain pathogenicity. In a chicken infection model, VGs belonging to *rfb* and *sse* operons showed differences and were linked with pathogenicity. The high-throughput, targeted NGS-based AmpliSeq_Salm_227VG_ procedure provided previously unknown information about variation in select virulence genes that can now be applied to a much larger population of *Salmonella* for evaluating pathogenicity of various serovars of *Salmonella* and for risk assessment of foodborne salmonellosis.

## 1. Introduction

Among members of the genus *Salmonella*, 227 virulence genes (VGs) have been described in the scientific literature, as the determinants of the four critical stages of *Salmonella* pathogenesis, namely: attachment, invasion, intramacrophage survival, and systemic dissemination [1,2]. Observations on the roles of VGs in *Salmonella* pathogenesis have mostly been obtained from experimental murine infection studies using *Salmonella* mutants, while only a few publications on pathogenesis have resulted from human NTS infection [3]. Despite some limitations, including result reproducibility [4,5], rodent models, in general, have formed the basis of the current understanding about the mechanism of action. Probably, a more important limitation of the mouse model is that it may not accurately mimic a human *Salmonella* infection. For instance, the serovar Typhimurium causes a typhoid-like illness in mice, but in the overwhelming number of human cases, it results in gastroenteritis. Many other models including monocytic macrophage cells from humans (U937 and THP-1; [6]) and non-macrophage cell lines from mice (RAW264.7) and humans (Caco-2 and INT-407; [7,8]), amoeba [8] and in vitro gastrointenstinal systems [9] have provided further illumination of *Salmonella* virulence. Collectively, many VGs have been well-characterized and proven to affect *Salmonella* behaviour and by extension the epidemiology of the disease. The application of genomics and bioinformatics has continued to shed increasing light on the biology of *Salmonella*, beyond that which can be obtained using experimental models and thus provide information that can be translated for the control of this rather important foodborne pathogen. To that end, the *Salmonella* Foodborne Syst-OMICS research database (SalFoS, https://salfos.ibis.ulaval.ca/, accessed on 30 December 2021), which was developed as part of a project on the diagnostics, surveillance, and control of *Salmonella,* contains a total of 3143 draft genomes of *Salmonella* (as of December 2021) and is a very useful repository of *Salmonella* genomes to identify biomarkers for surveillance and food outbreak investigations and to facilitate the development of tools to control salmonellosis [10]. In addition, the bioinformatics analysis of 500 *Salmonella* genomes completed by Rakov and colleagues (2019) led to the identification of 70 allelic variants of in silico expressed VGs associated with pathogenesis either manifesting as gastrointestinal or invasive disease [11]. Nevertheless, there still remains a gap in our current ability to infer the pathogenic potential or virulence of a strain based on the presence or absence of known virulence genes. Thus, as a first step in an attempt to interpret the clinical, epidemiological or experimental observations following *Salmonella* infection, the association between the VGs profile and the pathogenic potential of a strain needs to be established. This should help to embark on an overall assessment of the virulence potential of a strain of interest.

Despite the overall genetic similarity of *Salmonella enterica* organisms, vastly different diseases and distinct immune responses are elicited in humans by the different serovars [12]. To that end, comparative genomic analyses of multiple *Salmonella* serovars have conclusively identified genetic factors of the bacterium as an underlying cause of, or contributor to, the various disease manifestations caused by the pathogen [12]. For instance, the epidemiologically important NTS serovars linked with a high burden of foodborne *Salmonella* outbreaks in humans worldwide include Typhimurium, Enteritidis, Heidelberg, and Newport [13,14], yet a vast number of serotypes are rare causes of outbreaks [15] or were never incriminated in foodborne disease outbreaks in jurisdictions with wholesome public health records. Globally, a relatively small proportion of the thousands of known *Salmonella* serovars is associated with disease outbreaks in humans [16]. In this study, we sought to use a targeted NGS AmpliSeq^TM^ procedure by focusing on the 227 VGs of *Salmonella* recently described after a comprehensive evaluation of the scientific literature [1]. The assay was designed with the aid of 80 publicly available closed *Salmonella* genomes as references. We have demonstrated differences in the profiles of 227 VGs between strains and also displayed VGs variants of amplicons for certain field strains.

## 2. Materials and Methods

### 2.1. Salmonella Strains and Genomes

Eighty *Salmonella* reference genomes were downloaded from NCBI Batch Entrez (https://www.ncbi.nlm.nih.gov/, accessed on 15 October 2019; Table 1) and used to construct a primer set for the development of the AmpliSeq_Salm_227VG_ procedure which is a targeted sequencing method for detecting and characterizing virulence genes of *Salmonella*. The AmpliSeq_Salm_227VG_ procedure was applied to two groups of *Salmonella* organisms. The first group consisted of 35 strains retrieved from the pathogen inventory of the Ottawa Laboratory Fallowfield, Canadian Food Inspection Agency, and included 10 *Salmonella* Typhimurium strains of wild bird origin (see below—pathogenicity assessment of 10 *S.* Typhimurium strains in a chicken model). Phage typing of the organisms was carried out at the *Salmonella* Reference Centre, National Microbiology Laboratory, Public Health Agency of Canada, Guelph, ON, Canada. The second group was made of 34 *Salmonella* strains sourced from the SalFoS database (SalFoS; https://salfos.ibis.ulaval.ca/, accessed on 27 January 2020) [10], which was developed as part of the Genome Canada *Salmonella* Foodborne Syst-OMICS project and was provided by Dr. R. C. Levesque (Laval University, Quebec City, QC, Canada). The serovar designations of the strains belonging to the second group as well as the metadata of the organisms were not provided at the time of testing (blind testing), which allowed us to infer the serovar designation using the AmpliSeq_Salm_227VG_ procedure (see below), and the results were compared with the widely used *Salmonella* genome serovar designation tool—the *Salmonella* In Silico Typing Resource (SISTR) [17]. 

### 2.2. AmpliSeq_Salm_227VG_ Procedure

**Figure 1:**
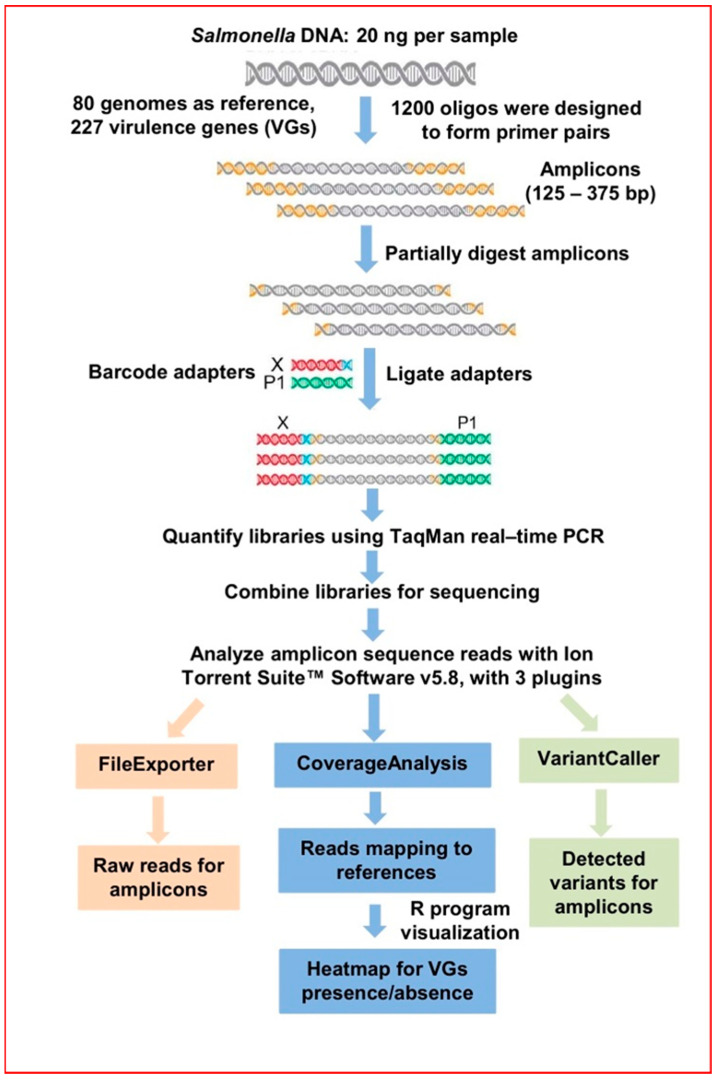
Workflow for the adaptation of AmpliSeq technology to develop and construct the AmpliSeq_Salm_227VG_ panel. VGs: virulence genes.

The AmpliSeq technology (Figure 1) is a targeted sequencing procedure of amplicons derived from ultra-high multiplexing of a large number of oligonucleotide primers that bind to areas of interest in a genome, which are then amplified and used to construct a DNA library [18,19]. We have employed the AmpliSeq technology to develop an assay which amplifies, determines the nucleotide sequences, and identifies DNA fragments coding for 227 *Salmonella* VGs. In designing our AmpliSeq assay for all known *Salmonella* VGs [5], which we term AmpliSeq_Salm_227VG_, sets of primers able to bind each virulence gene present in all the reference genomes were developed and pooled (ThermoFisher Scientific, South San Francisco, CA, USA). To that end, three principles were followed: (a) to increase coverage for the conserved and more variable VG sequences, multiple rounds of aligning at progressively different locations on each gene were carried out and the generated primers were pooled; (b) majority of the rounds of alignment focused on conserved areas but subsequently homology was relaxed to allow one single nucleotide change at the 5′ in up to half of primers with the aim of increasing coverage of targets with variable sequence; (c) stringent primer specificity filters were applied for the bulk of the design and the specificity parameters were relaxed in later rounds to increase coverage. Using our 80 reference genomes (see above under *Salmonella* strains and genomes) and 227 VGs, our expected pool primer would have ranged from 454 oligos (if all the VGs were completely conserved among the 80 genomes) to 36,320 oligos (if there was no conservation among all 227 genes, requiring the use of distinct primer pair for each of the VGs among all 80 genomes). Because of bioinformatics scan of target fragments to maximize the use of conserved areas of each VG across all 80 genomes, we identified a total of 1200 unique oligos capable of in silico generation of 16,729 amplicons (instead of a theoretical number of 18,160 amplicons based on 227 VG × 80 genomes); the expected amplicon sizes ranged from 125 bp to 375 bp. The degeneracy of the primer pool resulted in a large number of amplicons which was expected to contain all the variant forms within the 80 reference genomes (Table S1), and ensured that VGs would almost certainly be amplified and identified if present in a strain of *Salmonella.* Detailed information of the amplicons including size and start/stop locations are provided in Table S2.

### 2.3. Library Preparation and Sequencing

High-quality DNA was extracted using the Wizard genomic DNA purification kit (Promega, Madison, WI, USA) and assessed with a spectrophotometer (DU 730; Beckman Coulter, Mississauga, ON, Canada) and Qubit 2.0 fluorometer (Life Technologies, Carlsbad, CA, USA). Libraries were constructed with 20 ng of *Salmonella* DNA each using the Ion AmpliSeq library kit plus protocol (ThermoFisher, Mississauga, ON, Canada) and PCR amplified using the following parameters: primer pool, 20 ng of template DNA and 15 cycles of PCR, consisting of a denaturation step at 99 °C for 15 s and an annealing/extension step at 60 °C for 8 min. Following the PCR step, DNA amplicons were partially enzyme-digested and ligated with barcode adapters, according to manufacturer’s instructions. To enable sequencing of the different samples on a single Ion Torrent chip and save costs, the Ion Xpress™ Barcode kit was used to tag each of the 23 sample libraries. The barcoded libraries were quantified using the Ion library TaqMan quantification kit (Cat. No. 4468802) and were diluted to give a final template concentration of 100 pM. Enriched beads were prepared with the Ion OneTouch 2 System using the Hi-Q View OT2 Kit and sequencing was performed on the Ion Torrent Personal Genome Machine (PGM) machine using Ion PGM 400 Sequencing kit and an Ion 318™ chip. Sequence data were generated by the PGM (ThermoFisher, Waltham, MA, USA) over a period of 7 h.

### 2.4. Bioinformatic Analysis

**Figure 2:**
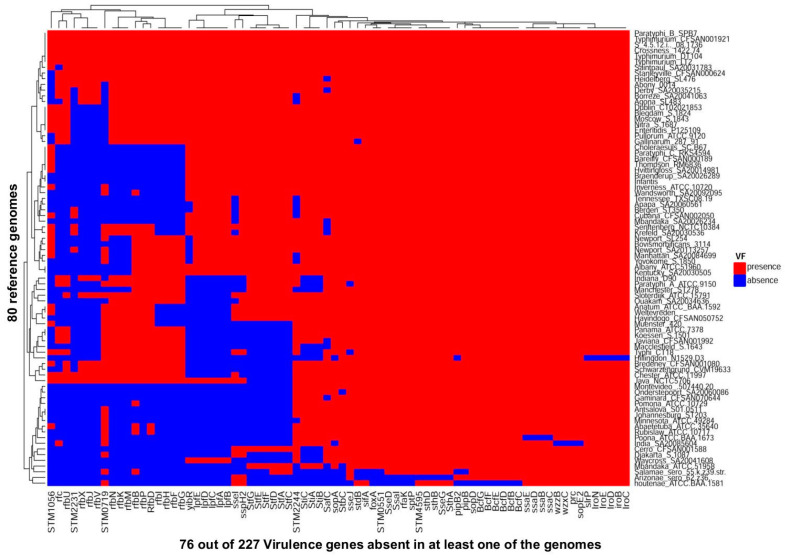
Distribution of 227 virulence genes in 80 reference *Salmonella* genomes used in designing the AmpliSeq_Salm_227VG_ panel. Red and blue colors represent gene presence and absence, respectively. The *x* axis contains the list of 76_80refs_ variable genes which are absent in at least one genome and the y axis shows the 80 *Salmonella enterica* reference genomes.

Sequence data analysis was automated as a streamlined NGS bioinformatics workflow using the Ion Torrent Suite™ Software v5.8 (ThermoFisher, Mississauga, ON, Canada) (Figure 1). Specifically, we used three plugins namely, FileExporter (version 5.12.0.0), CoverageAnalysis (version 5.12.0.0) and VariantCaller (version 5.12.0.4) with default parameters to generate VGs reads output which were transferred to an Excel document (Microsoft Corporation, Redmond, WA, USA) to illustrate the presence or absence of the 227 VGs and the variants for each *Salmonella* strain (Table S3). Positive reads indicating the detection of VGs in the AmpliSeq panel was designated “1”, whereas “0” indicated an absence (Table S4). The data were fed as inputs into R (RStudio, Boston, MA, USA), and four different R libraries consisting of ComplexHeatmap, magrittr, grid, and vegan were used to produce a heatmap depicting VG presence or absence (Figure 2).

### 2.5. Detection of Variants Using Ion Torrent Suite 

Customized Ion Torrent variant caller plugin (version 5.12.0.4) was used to detect variants among amplicons of VGs. For each strain, the Ion Torrent suite automatically mapped amplicon sequence to the most closely matching, pre-loaded reference genomes reads which is often the sequence that corresponded to the correct or assigned serovar (See Table 2 legend). Thereafter, sequence variation in each VG was detected by the variant caller by comparing the respective VGs leading to the identification of single nucleotide polymorphism (SNP), multiple nucleotide polymorphism (MNP), deletion (DEL) and insertion (INS) in each amplicon. The variants, serovar names, gene names, variants types and frequency of detected variants were compiled and summarized in an Excel table. Cut-off value for frequency was rigorously set at 90% to exclude spurious variants.

### 2.6. Pathogenicity Assessment of 10 S. Typhimurium Strains in a Chicken Model

All experiments involving animals were performed in accordance with Canadian Council for Animal Care guidelines and the experimental protocols were approved by the Canadian Food Inspection Agency Animal Care Committee (Approved Protocol ACC#08-06). Ten strains of *S.* Typhimurium used in our animal infection experiments were isolated from wild birds (sparrow, gull, cormorant and chicken) in Saskatchewan, Canada and were provided by Dr. Musangu Ngeleka of Prairie Diagnostic Services Inc. (Saskatoon, Saskatchewan, Canada). Frozen glycerol stocks of *Salmonella* were retrieved from storage, inoculated into Brain Heart Infusion (BHI) media and grown at 37 °C in a rotating shaker overnight. Bacteria counts in log phase culture were estimated by determining the optical density value at A_600_ and enumerated by plating inoculum used for animal infection on BHI plate agar followed by an overnight culture. Two-week-old white leghorn chicks were wing-banded and subcutaneously inoculated with 0.2 mL of *Salmonella* Typhimurium bacterial suspension containing ~1 × 10^8^ colony forming units. To avoid cross infection, the group of inoculated chicks exposed to the same inoculation dose was housed in a single isolator. Birds were inspected twice daily and weighed daily from the time of inoculation to the end of the experiment; clinical signs (lameness, ruffled feathers, loss of weight) and mortalities were recorded.

### 2.7. Pulsed-Field Gel Electrophoretic (PFGE) Analysis

The ten strains of *Salmonella* Typhimurium of wild bird origin selected for in vivo evaluation of intraserovar comparative pathogenesis experiment were subjected to PFGE using the restriction enzymes *Xba*I and *BIn*I according to the PulseNet Canada method “General Pulsed Field Gel Electrophoresis for *Escherichia coli*, *Salmonella* and *Shigella*”.

(http://www.pulsenetinternational.org/assets/PulseNet/uploads/pfge/PNL05_Ec-Sal-ShigPFGEprotocol.pdf, accessed on 19 June 2019). PFGE patterns were designated using the BioNumerics software v6.01 (Applied Maths, Sint-Martens-Latem, Belgium), after comparison to the PulseNet Canada database at the Public Health Agency of Canada’s National Microbiology Laboratory (Winnipeg, MB, Canada).

### 2.8. Statistical Analysis and Data Availability

The normal distribution of VGs was assessed using the statistical function in Excel (Microsoft Corporation). Result was judged significant if *p* < 0.05. Raw sequence reads of amplicons generated using PCR primers directed at 227 virulence genes for each of the 69 *Salmonella enterica* strains (see Table S4) and determined using the Ion Torrent PGM have been deposited in the NCBI databank under the BioProject Number PRJNA748885 (https://www.ncbi.nlm.nih.gov/bioproject/PRJNA748885, accessed on 1 January 2022). 

## 3. Results

### 3.1. Characteristics of 80 Salmonella Reference Genomes

Eighty *Salmonella enterica* genomes representing four subspecies and 75 serovars obtained from the GenBank were used as reference genomes to develop the AmpliSeq_Salm_227VG_ tool designed to evaluate the potential pathogenicity of *Salmonella* based on the presence/absence of 227 VGs (Table 1). Genome sizes ranged from 4,482,117 bp (serovar Pomona) to 5,395,280 bp (serovar India). The distribution of the VGs followed a normal distribution (mean = median = mode = 210 VGs; *p* = 1.6 × 10^−28^). In general, strains known to cause foodborne illnesses had an average or higher number of VGs. Serovars Enteritidis, Typhimurium, Heidelberg and Newport which are the commonest causes of foodborne illnesses had a high number of VGs (217–227). In contrast, the less virulent subspecies *houtenae*, *arizonae* and *salame* and the other serovars of subspecies *enterica* not commonly associated with foodborne illnesses tended to have fewer VGs (177–195). 

### 3.2. AmpliSeq_Salm_227VG_ Procedure for Evaluating Salmonella Virulence Genes 

The in silico simulation of the AmpliSeq_Salm_227VG_ on the 80 reference genomes led to the generation of VG targets presented as a heatmap that serves as a confirmation of the presence or absence of the 227 VGs in each of the 80 reference genomes (Figure 2). The results showed that 151 genes were present in all the genomes and are thus described as core_80ref_ VGs (data not shown in Figure 2). In contrast, the Variable_80refs_ VGs consisted of 76 genes, which were absent in at least one of the reference genomes. 

### 3.3. AmpliSeq_Salm_227VG_ to Detect the Target VGs in 35 Salmonella Field Strains 

**Figure 3:**
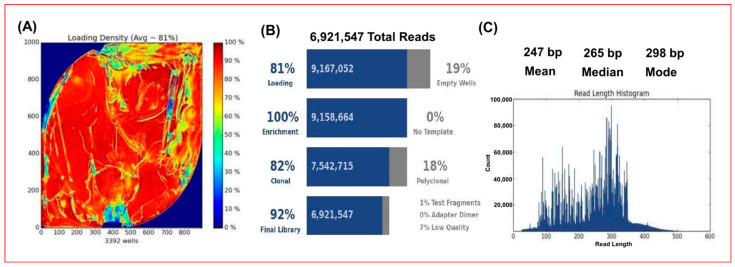
Analysis metrics for Ion Torrent Personal Genome Machine (PGM) amplicon sequencing for virulence genes present in a pool of 23 *Salmonella* strains. (**A**) Heatmap showing an optimal Ion Sphere Particle (ISP) density loading of 81% on the surface of PGM 318 chip. Red and blue colors represented the highest and lowest loading density areas, respectively. (**B**) A total of 6.9 million reads was obtained from the sequencing run and 82% reads were clonal. (**C**) Read length distribution of this representative run, with mean, median, mode length of 247 bp, 265 bp and 298 bp, respectively.

We evaluated the occurrence of the 227 VGs among the first set of 35 field strains of *S. enterica*, which belonged to 23 different serovars. A representative result of one sequencing run on PGM using a 318 chip is shown in Figure 3. The percentage of VGs amplicon reads mapping to a reference genome ranged from 96–99% (Table 2). Sequence reads from 31 of the 35 strains matched to the respective reference genome and allowed an accurate inference of the serovar of the organism. Two of the remaining four strains belonged to serovar Worthington for which there was no reference genome available at the start of our study. Consequently, the two strains matched to the closest serovar Mbandaka at 96% agreement, which was the least observed in this study (Table 2). These mismatches were highlighted, unsurprisingly, by an enormous number of variants (371 and 372). The two other mismatches involved the pair of serovars Enteritidis and Nitra, which are known to have genetic similarities [20] and surprisingly, serovars Gallinarum and Indiana (Table 2). For all 33 strains with a corresponding reference genome, the number of VSGs automatically deduced using the mapping procedure agreed well with the predicted VGs for the reference genome (Table 2). The AmpliSeq_Salm_227VG_ procedure also detected variants of amplicons between a test strain and its corresponding reference genome. The total number of variants in all the 29 tested strains, which mapped to their respective reference genomes are summarized in Table 3 and variant details including serovars, gene names, nucleotide changes, frequency and location are listed in Table S3. Out of the 227 VGs, 96 had variants. The serovar showing the highest number was *S.* Montevideo at 277 variants (Table 2 and Table 3). The six most variable genes were *safC*, *sseL*, *sseD*, *sseE*, *ssaK* and *stdB* having 26, 13, 11, 10, 10 and 10 variants, respectively (Table 3). Two of these genes, *safC* and *stdB*, are involved in attachment, while the remaining 4, namely *sseD*, *sseE*, *sseL* and *ssaK* are involved in intramacrophage survival of *Salmonella* (Table 4).

### 3.4. Virulence Genes among the Test Panel of 35 Salmonella enterica Strains

**Figure 4:**
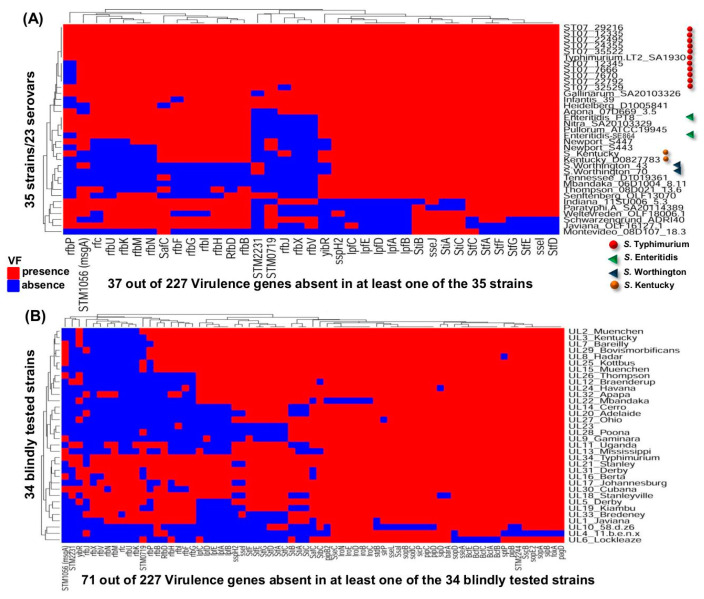
Heatmap illustration showing presence/absence of variable virulence genes (VGs) of *Salmonella*. (**A**) The 35 OLF strains representing 23 serovars. (**B**) The 34 SalFos strains. The *x* axis contains the list of variable VGs out of 227 used to design primers for the AmpliSeq_Salm_227VG_ tool. The *y* axis depicts the *Salmonella* strains and their identities. Red and blue colors represent presence and absence of a VG, respectively. The following serovars are highlighted because more than one strain was used in this study: Typhimurium ●, Enteritidis ▲, Worthington ▲, Kentucky ●. The remainder of the 227 VGs which were found in all strains (core VGs) are not shown in this illustration.

Among the 35 field strains tested with the AmpliSeq_Salm_227VaG_ procedure, 190 VGs were found to be present in all strains while 37 VGs (16%) were absent in at least one of the genomes (Variable_35OLF_ VGs = 37). Many of the variable genes belonged to five operons, namely *lpfABCDE*, *stfACDEFG*, *stiABC, sseIJ* and *rfbBDFGHIJKMNOUVX*. In particular, the *rfbP* (*n* = 16), *rfbV* (*n* = 17), *rfbJ* (*n* = 18) *rfbX* (*n* = 18) and *STM2231* (*n* = 19) were not detected at a high frequency. Phylogenetic analysis of the sequence reads showed that out of 35 strains which consisted of 23 serovars, two *S.* Kentucky, two *S.* Worthington and 11 *S.* Typhimurium strains tended to aggregate together, respectively, as shown by the presence of three distinct clusters (Figure 4A; see orange dot, blue triangle and red dot respectively). Thus, a strong serovar clustering occurred based on the presence or absence of the VGs in *Salmonella* strains. However, one Enteritidis strain (SE864) was noticeably separated from the other (PT8) by Nitra and Pullorum, and this was attributable to the loss of four *rfb* genes in Enteritidis_SE864 (Figure 4A).

### 3.5. AmpliSeq Analysis in the Validation Panel of 34 Salmonella Strains

**Figure 5:**
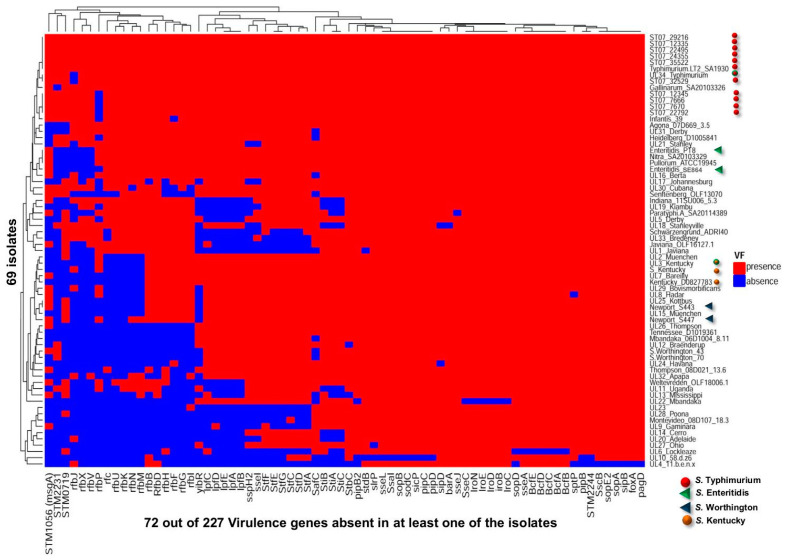
Heatmap illustration of reads presence/absence for 72 variable virulence genes (variable_69strains_ VGs) from a total of 69 *Salmonella* strains (35 OLF known and 34 SalFos strains). The *x* axis shows the 72 variable_69strains_VGs while the y axis represents all the tested 69 *Salmonella* strains. Red and blue colors represent presence and absence of a VG, respectively. For annotation purpose: Typhimurium ●, Enteritidis ▲, Worthington ▲, Kentucky ●. The illustration is a pool of the data from Figure 4A,B to show the clustering of the *Salmonella* serovars based on analysis of VGs sequences.

To validate the AmpliSeq procedure, we comprehensively examined another group of *Salmonella* strains made up of 34 organisms retrieved from the SalFoS database, but provided to the laboratory without the disclosure of any metadata regarding identity or virulence of the organisms (Table 5). Following blind analysis using the AmpliSeq_Salm_227VG_ procedure, the results were analyzed incorporating the metadata especially the serovar designations which were subsequently provided by the SalFos database coordinator. The heatmap displayed the presence/absence of VGs and served as an illustration of the overall pattern as well as the diversity of the VGs distribution among these 34 SalFoS analyzed strains/serovars (Figure 4B). A total of 71 genes or 31% of 227 genes was absent in at least one of the strains (Variable_34SalFoS_ VGs = 71). Because the AmpliSeq results obtained for the 34 SalFoS strains were comparable to those obtained for the initial batch of 35 OLF known strains, we combined the presence/absence profiles for all 69 strains as shown in Table S4. Phylogenetic analysis resulted in the clustering of the serovars (Figure 5). Three different serovars—Typhimurium (*n* = 12; red dot), Kentucky (*n* = 3; orange dot), Worthington (*n* = 2; blue triangle)—tended to aggregate into three distinct clusters, respectively (Figure 5), which agreed with results from Figure 4A. The only two strains from a group of 34 SalFoS (i.e., UL3_Kentucky and UL34_Typhimurium, which shared the same serovars as other strains from the group of 35 OLF known strains, were precisely clustered in their expected groups namely, *S.* Kentucky and *S.* Typhimurium, respectively (Figure 5, either red or orange dot with green border). For the different pathogenesis stages, the 155 Core_69_strains_ VGs were made of 25, 54, 32 and 44 genes for the attachment, invasion, intramacrophage survival and systemic dissemination steps, respectively. The 72 Variable_69_strains_ VGs included 24, 19, 7 and 22 genes in the attachment, invasion, intramacrophage survival and systemic dissemination stages, respectively (Table 6).

### 3.6. Pathogenicity Evaluation for 10 OLF Salmonella Typhimurium Strains in a Chicken Infection Model

**Figure 6:**
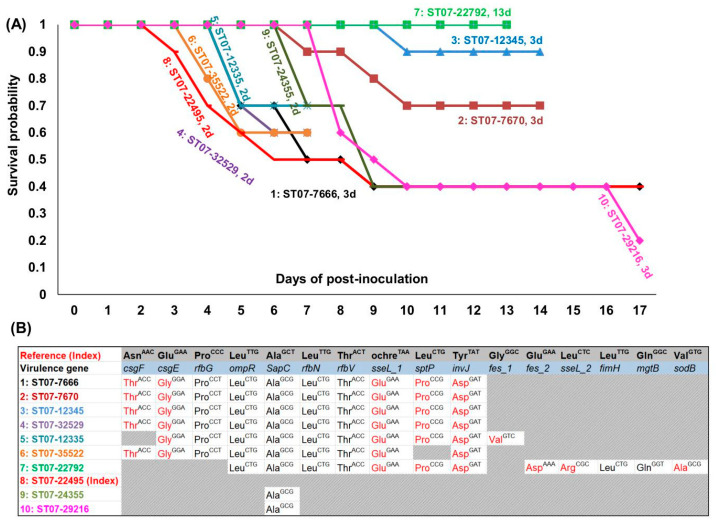
Survival rate plot of chickens inoculated with *Salmonella* Typhimurium strains and virulence gene variants detected among the strains, *n* = 10. (**A**) Survival rate plot of chicken inoculated with 10 *S.* Typhimurium strains. The sample name is labeled with ID: strain name, day post-infection when clinical signs were first observed, for instance, the number 1 sample was labeled as “1: ST07-7666, 3d”; the sample name was highlighted with the same color as its survival plot line color. The most virulent strain was ST07-22495 No. 8 strain (red color line with red short horizontal line highlighted) whereas the least virulent strain was No. 7 ST07-22792 strain (green color line with green square highlighted). (**B**) Detected variants among the 10 *S.* Typhimurium strains used for the infection experiment, using No. 8_ST07-22495 as index. For easy identity, the same color was used for strain names as well in panel (**A**). The variants causing non-synonymous amino acid changes are highlighted in red.

Selected from the 69 strains tested for their VGs profiles (Figure 3A and Figure 5), 10 *Salmonella* Typhimurium strains were used to examine pathogenicity in a chicken model to assess virulence at the intra-serovar level by observing clinical signs and survival (Table 7) [21]. Following subcutaneous inoculation of chickens, birds inoculated with ST07_22495 (No. 8) showed the highest pathogenicity (lowest survival rate of 40% and earliest clinical signs at 2 dpi), whereas chickens inoculated with strain ST07_22792 (No. 7) displayed the least pathogenicity (all survived at 13 days post inoculation) (Figure 6A). The two strains with distinct pathogenicity attributes also showed contrasting properties in terms of host, phage typing and PFGE patterns (Table 7, Figure S1).

A comparison of the full complement of the VGs of the most pathogenic strain (ST07-22495) with the other nine Typhimurium strains revealed varying degree of genetic divergence which correlated with pathogenicity (Figure 6A,B and Table 7 and Table S5). Two strains (ST07_24355, ST07_29216) which were the least divergent from the most pathogenic strain had only 1 synonymous variant, i.e., no amino acid change. These three most pathogenic strains shared an ochre mutation which inserted a stop codon in the *sseL*, a VG involved in intramacrophage survival of *Salmonella*, resulting in a truncated open reading frame of 309 amino acids. This mutation was not found in any other strain as the remaining strains including the least pathogenic had a *sseL* gene coding for the entire 340 amino acids. The three least diverged strains (i.e., with no amino acid change among the VGs—ST07-22495, ST07_24355, ST07_29216) showed the highest pathogenicity as shown by fewer chickens surviving infection and most severe clinical signs (lameness, ruffled feathers, loss of weight). The strain bearing sequences with the next degree of similarity to the most pathogenic strains (i.e., ST07-35522, 9 variants) also showed a degree of pathogenicity next to the three most pathogenic strains as measured by a combination of mortality and severity of clinical signs among survivors, especially lameness. In contrast, the strain most genetically divergent from the reference strain (namely, ST07-22792) had 17 loci with single nucleotide changes that were different from the most pathogenic strain. The most divergent strain was very clearly the least pathogenic and the only one in which none of the inoculated chickens died during the time monitored. Of the remaining 6 strains whose divergence lie between the three fairly uniform strains on the basis of genetics and virulence on one hand (i.e., ST07-22495, ST07_24355, ST07_29216) and the most divergent and least pathogenic strain (ST07-22792) on the other hand, one strain with a genetic variation closer to the three pathogenic strains (ST07-35522, Figure 6B) had 9 variant loci consisting of both synonymous and non-synonymous mutations. This strain was also the fourth most virulent strain after the earlier described three most virulent strains (Figure 6A). The middle group of five strains had 10 variant loci made up of synonymous and non-synonymous changes when compared to ST07_22495 and all the strains had the milder disease (Figure 6A,B and Table S5). One of the strains (ST07-12335) had a unique variation in the *fes* gene and behaved in a slightly more virulent fashion that the other 4 strains which had a very uniform variation at the 10 loci which did not include the *fes* gene. However, it was not as virulent as the three most pathogenic strains described above (ST07-22495, ST07_24355, ST07_29216) or the one strain (ST07-35522) that followed them in virulence behavior and genetics. In all, these observations clearly illustrate that genetic differences among the VGs can be linked to the varying degrees of pathogenesis in the serovar Typhimurium which generally tended to be pathogenic.

## 4. Discussion

The functions and regulations of a restricted number of VGs have been historically studied in a limited number of serovars mainly Typhimurium and Enteritidis [2,3,11]. Consequently, the overall contribution of the VGs to pathogenesis in *Salmonella*, either in the well-studied serovars or the others remains elusive [22]. This study used a targeted AmpliSeq_Salm_227VG_ procedure to interrogate *Salmonella* strains for the purpose of determining the presence of up to 227 VGs and their sequence variation. The approach is analogous to carrying out hundreds of PCR reactions for each strain and sequencing each amplicon. Achieving these steps by simultaneous analysis of many strains at the same in a single assay provides an unprecedented power backed by automated bioinformatics analysis. The same objective can be achieved by extracting and analyzing the VGs present in an available, good-quality whole genome sequence however the AmpliSeq_Salm_227VG_ procedure is targeted and therefore faster, with a higher throughput and the bioinformatics analysis is automated and can therefore be carried out by an analyst will minimal bioinformatics skills, given that no command line procedure is required. We compiled our list of 227 genes following a rigorous literature review. *Salmonella* virulence has been the focus of many studies and many genes are known to contribute to *Salmonella* pathogenicity [13,23]. However, the vast majority of studies typically analyze a few genes [24,25,26] and occasionally, a few dozen genes [11], to evaluate the ability of the organisms to cause disease. The only study that we are aware of that evaluated a high number of VGs used 219 genes, from a total of 233 of which 14 were absent among 59 *Salmonella* strains [27]. Because of its extracellular and intracellular phases, it is not surprising that *Salmonella* has many more virulence genes than other enteric bacteria such a *Escherichia coli* which may have fewer than 100 VGs [28,29]. Even though *Salmonella* and *E. coli* both colonize the same part of the gastrointestinal tract, have similar genome sizes and cause fairly similar disease phenotype, i.e., gastroenteritis, *E. coli* only has an extracellular phase while *Salmonella* inhabits both extracellular and intracellular locations and will need more VGs to survive and thrive. Despite our effort to identify as many *Salmonella* VGs as possible, our list is probably not exhaustive as more virulence genes are observed with new studies. At the onset of our investigation, we established the sequences of the 227 VG among 80 reference genomes which represented a diverse set of epidemiologically important serovars. We tested our hypothesis that the higher the number of VGs present in a *Salmonella* strain the greater its ability to cause disease in humans and found that representative genomes of serovars responsible for frequent outbreaks, such as Enteritidis, Typhimurium, Heidelberg and Newport, tended to have higher number of VGs. 

We were also interested in evaluating the distribution of the VGs among the strains studied and found 151 of 227 VGs or 66.5% (core_80refs_ VGs) to be present in all the 80 reference genomes (Figure 2). The predominance of the core VGs among *Salmonella* serovars was further supported by the insightful results of tests conducted on 34 *Salmonella* strains (Figure 4B). The occurrence of a majority of VGs in many *Salmonella* strains including those that do not normally cause infections in humans suggest that the variability in the pathogenic potential of *Salmonella* strains may be attributed to the variable segment of the VGs. We demonstrated an association between variable VGs and distinct phases of *Salmonella* results as shown by preponderant variation among operons and genes playing a role in the attachment (*lpf*, *stf* and *sti* operons), invasion *(STM2231*), intramacrophage survival (*sse* operon) and systemic dissemination (*rfb* operon). The use of 80 reference genomes which included the most commonly reported serovars contaminating food, was adequate for the AmpliSeq_Salm_227VG_ tool to furnish an insight in the distribution of VGs in all tested *Salmonella* strains. Even when only 18 of the 34 tested strains had matching serovars among the 80 reference genomes (Figure 4B), information obtained on the distribution of VG and variants among all strains was adequate to evaluate their potential pathogenicity. 

A chicken infection model further shed light on the effect of the *rfb* operon in virulence. Among the 10 *S.* Typhimurium strains used in experimental infections, five strains (ST07_29216, ST07_12335, ST07_22495, ST07_24355, and ST07_35522) possessed all the 227 VGs and a full complement of the *rfb* operon and demonstrated the highest virulence based on mortality or short incubation period in infected chickens (Figure 6A). The nucleotide variations among three of the five strains were very similar and the remaining two strains showed a different pattern of variation which could be distinguished from the other, five less pathogenic strains (Figure 6B). The less pathogenic strains did not have a full complement of the *rfb* operon and were missing the *rfbP* gene (ST07_12345, ST07_7670, and ST07_22792) or the *rfbJ* gene (ST07_32529) (Figure 4A and Figure 5). One strain, ST07_7666, was missing the *rfbP* gene but behaved as an outlier combining symptoms associated with low virulence as the other non-pathogenic strains (delayed onset of clinical symptoms at day 3) but with reduced survival similar to the pathogenic strains (Table 7 and Figure 6A). The behaviour of strain ST07-7666 is most likely influenced by other yet to be identified genes. Two additional Typhimurium strains not used for experimental infection either had a full *rfb* operon (LT2) or was missing *rfbJ* (UL34). We found that the *sseL* gene, which codes for a deubiquitinase and contributes to pathogenesis by macrophage killing [30] was similarly truncated among the three most pathogenic Typhimurium strains but had a full-length version among the remaining less pathogenic Typhimurium strains. How a truncated deubiquitinase may cause increased virulence is not clear but may be similar to other observations where a truncation is required for full protein activity [31] or increased enzymatic activity [32].

The least pathogenic *S.* Typhimurium strain (ST07_22792; nil clinical signs and mortality for 13 days post infection) was not only devoid of a full *rfb* operon but showed a very marked nucleotide variation and the greatest divergence, i.e., 17 single nucleotide changes, from the most pathogenic strains (ST07_22495; first clinical sign at 2 days post infection and 40% mortality at 13 dpi; Figure 6A and Table 7 and Table S5). Sequence divergence among strains belonging to the Typhimurium serovar mirrors published observation on the serotype Montevideo where marked variations were found in VGs belonging to different clades [33]. Further attributes of the avirulent ST07-22792 highlighted its distinct behaviour, namely: it is the only strain from a cormorant used in the study, had a unique phage type, 1, as well as a unique PFGE pattern for both primary and secondary enzymes (Table 7). Unlike the observations of Kuijpers and colleagues [27], we observed a serovar-based clustering of the *Salmonella* strains dictated by the VGs detected with using the AmpliSeq_Salm_227VG_ procedure (Figure 5). Our observation suggests that the VGs analysis may have field relevance: nucleotide sequences of the same target of more related organisms will be expected to show a higher degree of similarities compared to less related organism (e.g., different serovars). At the same time, the AmpliSeq results have provided a very high resolution of the differences among members of a serovar that is usually pathogenic based on the presence/absence of specific VG and VG variants. We previously reported a robust experimental chicken infection model used to demonstrate differences in the virulence of strains of *Salmonella* Typhimurium strains [21], and which was similar to that used by other workers to evaluate virulence for *Salmonella* Enteritidis [34]. We were able to use the infection model to demonstrate very convincingly that variations among VG translated to different virulence potential in vivo. 

The presence of 155 core_69strains_ VGs distributed over the four phases of *Salmonella* pathogenesis in all the strains tested (Table 6), including *Salmonella* organisms known to have low virulence and no or rare report of human infections [3], suggests that this set of genes may not contribute markedly to clinical outcomes in a “normal” human population. This perspective is reinforced by the observations that only a small proportion of *Salmonella* serovars—perhaps as few as 100 serovars—is responsible for a high proportion of foodborne human outbreaks. Yet, our observations from this study suggest that the core VG may well be present in all *Salmonella* serovars. We suggest that the core genes could confer virulence in an immunologically compromised, vulnerable population who may be susceptible to a large number of serovars [35]. Put differently, it appears that all *Salmonella* strains carry an inherent but not commonly expressed ability to be pathogenic, in which case the risk management of *Salmonella* contamination in food among specific demographics, especially consumers that are advanced in age may require a more rigorous oversight than presently done.

In conclusion, we have demonstrated a targeted NGS-based AmpliSeq procedure to simultaneously assess the presence/absence of 227 VGs and variants of amplicons in *Salmonella* in a high-throughput way. Focus on the variable VG could help infer risk posed by a *Salmonella* strain including a prediction of the ability of the organism to cause a foodborne outbreak and the severity of such an incidence.

## 5. Conclusions

*Salmonella enterica* is a common bacterial contaminant of food, however, only a relatively small number of serovars of the organisms are associated with disease outbreaks in humans, despite the description of a large number of virulence genes in the species. A comprehensive tool to evaluate the virulence potential of *Salmonella enterica* could provide an objective risk assessment of *Salmonella* contamination in food. We developed a sequence-based AmpliSeq_Salm_227VG_ tool for the simultaneous interrogation of many *Salmonella* isolates for the presence/absence of all the virulence genes. We demonstrated the presence of core virulence genes, detectable in all organisms tested, and variable virulence genes which appeared important in conferring the disease-causing status. Using in vitro experiments and bioinformatics tools, this study revealed a number of important variable virulence genes that play critical roles in the attachment (*lpf*, *stf* and *sti* operons), invasion *(STM2231*), intramacrophage survival (*sse* operon), and systemic dissemination (*rfb* operon) of *Salmonella*.

## Figures and Tables

**Table 1:** *Salmonella* reference genomes used in the design of AmpliSeq_Salm_227VG_ panel and their distribution of 227 known virulence genes.

ID	Subspecies/Serovar	^a^ Strain	Size (bp)	^b^ VGs (Max = 227)
CP006602.1	4,[5],12:i:-	08-1736	4,822,189	227
CP007532.1	Abaetetuba	ATCC 35640	4,547,600	199
CP007534.1	Abony	0014	4,737,447	225
CP001138.1	Agona	SL483	4,798,660	222
CP019177.1	Albany	ATCC 51960	4,805,448	216
CP007531.1	Anatum	ATCC BAA-1592	4,706,101	211
CP019116.1	Antsalova	S01-0511	4,648,086	196
CP019403.1	Apapa	SA20060561	4,801,658	206
CP006693.1	*arizonae 62:z36:-*	RKS2983	4,574,846	177
CP006053.1	Bareilly	CFSAN000189	4,730,612	210
CP019405.1	Bergen	ST350	4,801,835	207
CP019406.1	Blegdam	S-1824	4,693,979	222
CP019407.1	Borreze	SA20041063	4,777,558	223
HF969015.2	Bovismorbificans	3114	4,680,283	216
CP022490.1	Braenderup	SA20026289	4,734,880	209
CP007533.1	Bredeney	CFSAN001080	4,603,849	211
CP012833.1	Cerro	CFSAN001588	4,651,400	200
CP019178.1	Chester	ATCC 11997	4,660,922	214
AE017220.1	Choleraesuis	SC-B67	4,755,700	210
CP019408.1	Crossness	1422-74	4,847,468	227
CP006055.1	Cubana	CFSAN002050	4,977,480	209
CP022494.1	Derby	SA20035215	4,850,334	223
CP019409.1	Djakarta	S-1087	4,668,861	195
CP001144.1	Dublin	CT_02021853	4,842,908	222
NC_011294.1	Enteritidis	P125109	4,685,848	222
NC_011274.1	Gallinarum	287/91	4,658,697	220
CP024165.1	Gaminara	CFSAN070644	4,801,841	194
CP017719.1	Hayindogo	CFSAN050752	4,765,719	210
CP001120.1	Heidelberg	SL476	4,888,768	225
CP019410.1	Hillingdon	N1529-D3	4,618,056	194
CM001471.1	*houtenae*	ATCC BAA-1581	4,672,567	175
CP022503.1	Hvittingfoss	SA20014981	4,940,239	210
CP022015.1	India	SA20085604	5,395,280	192
CP022450.1	Indiana	D90	4,779,514	213
LN649235.1	Infantis	NA	4,710,675	209
CP019181.1	Inverness	ATCC 10720	4,865,682	211
LT571437.1	Java	NCTC5706	4,756,780	221
CP004027.1	Javiana	CFSAN001992	4,634,161	207
CP019411.1	Johannesburg	ST203	4,651,794	196
CP022500.1	Kentucky	SA20030505	4,782,363	216
CP019412.1	Koessen	S-1501	4,566,169	208
CP019413.1	Krefeld	SA20030536	4,942,273	216
CP022117.1	Macclesfield	ST-1643	4,822,139	202
CP019414.1	Manchester	ST278	4,532,753	204
CP022497.1	Manhattan	SA20084699	4,732,484	216
CP019183.1	Mbandaka	ATCC 51958	4,905,181	198
CP022489.1	Mbandaka	SA20026234	4,796,292	207
CP019184.1	Minnesota	ATCC 49284	4,592,393	195
CP007530.1	Montevideo	507440-20	4,694,375	195
CP019415.1	Moscow	S-1843	4,690,402	222
CP019201.1	Muenster	420	4756014	203
NC_011080.1	Newport	SL254	4,827,641	217
CP019404.1	Newport	SA20113257	4,849,139	218
CP019416.1	Nitra	S-1687	4,691,807	222
CP022034.1	Onderstepoort	SA20060086	4,774,926	194
CP022116.1	Ouakam	SA20034636	4,874,915	212
CP012346.1	Panama	ATCC 7378	4,555,576	208
CP000026.1	Paratyphi A	ATCC 9150	4,585,229	210
CP000886.1	Paratyphi B	SPB7	4,858,887	227
CP000857.1	Paratyphi C	RKS4594	4,833,080	210
CP019186.1	Pomona	ATCC 10729	4,482,117	196
CP019189.1	Poona	ATCC BAA-1673	4,876,720	192
CP012347.1	Pullorum	ATCC 9120	4,694,842	221
CP019192.1	Rubislaw	ATCC 10717	4,572,929	198
CP022491.1	Saintpaul	SA20031783	4,775,303	226
CP022139.1	*salamae 55:k:z39*	NA	4,859,044	187
CP001127.1	Schwarzengrund	CVM19633	4,709,075	210
LN868943.1	Senftenberg	NCTC10384	3,746,274	NA
CP012349.1	Sloterdijk	ATCC 15791	4,817,791	218
CP017723.1	Stanleyville	SARB61	4,888,463	226
CP007505.1	Tennessee	TXSC_TXSC08-19	4,864,410	207
CP006717.1	Thompson	RM6836	4,707,648	210
NC_003198.1	Typhi	CT18	4,809,037	207
HF937208.1	Typhimurium	DT104	4,933,631	227
NC_003197.2	Typhimurium	LT2	4,857,450	227
CP006048.1	Typhimurium var. 5-	CFSAN001921	4,859,931	227
CP019417.1	Wandsworth	SA20092095	4,916,040	211
CP022138.1	Waycross	SA20041608	4,812,886	198
LN890520.1	Weltevreden	NA	5,129,845	211
CP019418.1	Yovokome	S-1850	4,640,929	215

^a^ NA: not available; ^b^ VGs = virulence genes.

**Table 2:** Results of AmpliSeq analysis performed on strains of *Salmonella* with known serovar designation.

Serovars/Strains	Predicted No.	Detected No.	Mapped Reads	Total Reads	^a^ On Target	Assigned Serovar	^b^ Variant Number
Agona 07D669 3-5	222	224	331,304	337,261	0.98	Agona	1
Enteritidis SE864	222	221	306,812	312,339	0.98	Enteritidis	8
Enteritidis PT8	222	222	313,320	319,143	0.98	Enteritidis	7
Gallinarum SA20103326	220	226	285,325	290,275	0.98	Indiana	28
Heidelberg D1005841	225	224	298,529	305,742	0.98	Heidelberg	0
Indiana 11SU006 5-3	213	217	330,510	335,680	0.98	Indiana	1
Infantis 39	209	226	276,145	282,107	0.98	Infantis	4
Javiana OLF16127-1	207	211	292,639	298,829	0.98	Javiana	47
Kentucky D0827783	216	215	278,679	283,095	0.98	Kentucky	2
Mbandaka 06D1004 8-11	207	208	257,337	264,129	0.97	Mbandaka	1
Montevideo 08D107 18-3	195	196	310,953	317,627	0.98	Montevideo	277
Newport S443	211	216	254,846	258,566	0.99	Newport	21
Newport S447	218	219	301,846	308,710	0.98	Newport	23
Nitra SA20103329	222	222	260,241	265,458	0.98	Enteritidis	390
Paratyphi A SA20114389	210	214	313,073	318,553	0.98	Paratyphi A	5
Pullorum ATCC19945	221	222	283,317	288,600	0.98	Pullorum	2
S.Kentucky	216	217	273,264	279,534	0.98	Kentucky	1
S.Typhimurium 07-12345	227	226	248,137	252,351	0.98	Typhimurium	11
S.Typhimurium07-12335	227	227	257,763	264,195	0.98	Typhimurium	11
S.Typhimurium07-22495	227	227	333,463	342,427	0.97	Typhimurium	15
S.Typhimurium07-22792	227	227	292,454	300,721	0.97	Typhimurium	5
S.Typhimurium07-24355	227	227	344,674	355,565	0.97	Typhimurium	14
S.Typhimurium07-29216	227	227	258,620	263,517	0.98	Typhimurium	14
S.Typhimurium07-32529	227	226	263,450	271,411	0.97	Typhimurium	11
S.Typhimurium07-35522	227	227	305,560	313,980	0.97	Typhimurium	11
S.Typhimurium07-7666	227	226	289,621	298,397	0.97	Typhimurium	11
S.Typhimurium07-7670	227	226	294,378	302,460	0.97	Typhimurium	11
S.Worthington_43	209	209	303,102	314,934	0.96	Mbandaka	371
S.Worthington_70	209	209	304,260	317,665	0.96	Mbandaka	372
Schwarzengrund ADRI40	210	212	290,699	295,369	0.98	Schwarzengrund	3
Senftenberg OLF13070	217	217	304,695	313,491	0.97	Senftenberg	-
Tennesse D1019361	207	209	291,490	298,976	0.97	Tennesse	1
Thompson 08D021 13-6	210	213	292,588	297,576	0.98	Thompson	0
Typhimurium LT2 SA1930	227	227	278,222	284,938	0.98	Typhimurium	0
Weltevreden OLF18006-1	211	213	280,421	286,778	0.98	Weltevreden	0

^a^ On target: the proportion of sequenced amplicon reads mapping to the target genes in the reference genome. ^b^ Two rounds of reads mapping were performed for the amplicons of each strain. First round of mapping was an automated real time alignment of sequence reads to the preloaded 80 *Salmonella* reference genomes resulting in serovar identification from the highest reads match. The identified serovar is depicted as the “Assigned Serovar” in this table. Subsequently, using the “Assigned Serovar” genome as reference, the second round amplicon reads mapping was carried out to find the number of variants present between the tested strain (listed in column named “Serovars/strains”) and its respective reference genome. Except for the serovar Montevideo, high variant number was predictive of wrong serotype designation and could be used as a means of assessing suspicious results. For instance, during the 1st round mapping, sequence reads from Worthington, Nitra and Gallinarum incorrectly mapped to the serovars Mbandaka, Enteritidis and Indiana, respectively and showed relatively high to very high number of variants.

**Table 3:** Number of virulence gene variants present in each of 29 *Salmonella enterica* strains identified with the correct serovar designation as a result of automated, real time mapping of sequence reads to preloaded reference genomes by the Ion Torrent sequencer.

Gene	No.	Gene	No.	Gene	No.	Gene	No.	Gene	No.	Gene	No.
*safC*	26	*pagC*	5	*fhuE*	2	*ymdA*	2	*fhuC*	1	*sapB*	1
*sseL*	13	*StiC*	5	*invA*	2	*barA*	1	*fimD*	1	*sapC*	1
*sseD*	11	*sptP*	4	*iroB*	2	*BcfE*	1	*fimF*	1	*sicA*	1
*ssaE*	10	*invJ*	3	*lpfA*	2	*BcfF*	1	*hilC*	1	*sifA*	1
*ssaK*	10	*mgtA*	3	*lpfC*	2	*crp*	1	*hofB*	1	*sipC*	1
*stdB*	10	*ompR*	3	*pagD*	2	*csgB*	1	*orgB*	1	*smpB*	1
*cirA*	9	*rfbN*	3	*rfaY*	2	*csgC*	1	*pipC*	1	*ssaJ*	1
*csgE*	9	*rfbV*	3	*rpoE*	2	*csgD*	1	*rfaB*	1	*ssaM*	1
*fimH*	9	*sipA*	3	*ssaI*	2	*csgG*	1	*rfaP*	1	*SseA*	1
*hilD*	9	*StbC*	3	*ssaO*	2	*entA*	1	*rfbM*	1	*stfF*	1
*iacP*	9	*fepD*	2	*ssaT*	2	*entC*	1	*rfbX*	1	*stfG*	1
*rfbG*	6	*fepG*	2	*sseI*	2	*fepE*	1	*rpoS*	1	*sthA*	1
*csgF*	5	*fes*	2	*sspH2*	2	*fhuB*	1	*sapA*	1	*ybdB*	1
*SafC*	26	*pagC*	5	*fhuE*	2	*ymdA*	2	*fhuC*	1	*sapB*	1
*sseL*	13	*stiC*	5	*invA*	2	*barA*	1	*fimD*	1	*sapC*	1
*sseD*	11	*sptP*	4	*iroB*	2	*bcfE*	1	*fimF*	1	*sicA*	1

**Table 4:** Virulence genes involved in the four known steps of *Salmonella* pathogenesis.

VG Type	No.	Gene Names
Attachment	49	*barA*, *bcfA*, *bcfB*, *bcfC*, *bcfD*, *bcfE*, *bcfF*, *bcfG*, *csgA*, *csgB*, *csgC*, *csgD*, *csgE*, *csgF*, *csgG*, *fimC*, *fimD*, *fimF*, *fimH*, *FimI*, *fimW*, *FimY*, *FimZ*, *hofB*, *hofC*, *lpfA*, *lpfB*, *lpfC*, *lpfD*, *lpfE*, *ppdD*, *safC*, *stbC*, *stdB*, *stfA*, *stfC*, *stfD*, *stfE*, *stfF*, *stfG*, *sthA*, *sthB*, *sthD*, *stiA*, *stiB*, *stiC*, *STM0551*, *STM4595*, *yibR*
Invasion/intracellular survival	73	*crp*, *hilA*, *hilC*, *hilD*, *hnr*, *iacP*, *iagB*, *invA*, *invB*, *invC*, *invE*, *invF*, *invG*, *invH*, *invI*, *invJ*, *ompR*, *orgA*, *orgB*, *orgC*, *pagC*, *pagD*, *pagP*, *pipB*, *pipB2*, *pipC*, *pipD*, *prc*, *prgH*, *prgI*, *prgJ*, *prgK*, *rpoE*, *rpoS*, *sapA*, *sapB*, *sapC*, *sapD*, *sapF*, *sicA*, *sicP*, *sifA*, *sipA*, *sipB*, *sipC*, *sipD*, *slrP*, *slyA*, *sodA*, *sodB*, *sodC*, *sopA*, *sopB*, *sopD*, *sopD2sopE2*, *spaO*, *spaP*, *spaQ*, *spaR*, *spaS*, *sprB*, *sptP*, *sspH2*, *STM1056 (msgA)*, *STM2231*, *STM2244*, *STM4315 (rtsA)*, *yejA*, *yejB*, *yejE*, *yejF*, *ymdA*
Intramacrophage survival	39	*csrA*, *hfq*, *mgtA*, *mgtB*, *mgtC*, *smpB*, *ssaB*, *ssaC*, *ssaD*, *ssaE*, *ssaG*, *ssaH*, *ssaI* *ssaJ*, *ssaK*, *ssaL*, *ssaM*, *ssaN*, *ssaO*, *ssaP*, *ssaQ*, *ssaR*, *ssaS*, *ssaT*, *ssaU*, *ssaV*, *sscA*, *sscB*, *sseA*, *sseB*, *sseC*, *sseD*, *sseE*, *sseF*, *sseG*, *sseI*, *sseJ*, *sseL*, *STM1410 (ssaX)*
Dissemination	66	*cirA*, *entA*, *entB*, *entC*, *entD*, *entE*, *entF*, *fepA*, *fepB*, *fepC*, *fepD*, *fepE*, *fepG*, *fes*, *fhuA*, *fhuB*, *fhuC*, *fhuD*, *fhuE*, *foxA*, *fruR*, *fUR*, *iroB*, *iroC*, *iroD*, *iroE*, *iroN*, *msbA*, *msgA*, *rfaB*, *rfaC*, *rfaD*, *rfaF*, *rfaG*, *rfaH*, *rfaI*, *rfaJ*, *rfaK*, *rfaL*, *rfaP*, *rfaQ*, *rfaY*, *rfaZ*, *rfbB*, *rfbD*, *rfbF*, *rfbG*, *rfbH*, *rfbI*, *rfbJ*, *rfbK*, *rfbM*, *rfbN*, *rfbP*, *rfbU*, *rfbV*, *rfbX*, *rfc*, *STM0719*, *udhA*, *wzxC*, *wzxE*, *wzzB*, *wzzE*, *ybdA*, *ybdB*
Total	227	

Where virulence genes are also known by alternate names, these are indicated in parenthesis, namely: *STM1056* (*msgA*), *STM4315* (*rtsA*), and *STM1410* (*ssaX*).

**Table 5:** Validation of AmpliSeq_Salm_227VG_ based on blind assessment of virulence and serovar designation for 34 *Salmonella* strains.

Strain ID	SalFoS ID	Serovar	^a^ VGs No.
UofLaval23	S158	NA	196
UofLaval4	S785	11:b:e,n,x	182
UofLaval10	S774	58:d:z6	183
UofLaval20	S361	Adelaide	199
UofLaval32	S1393	Apapa	214
UofLaval7	S603	Bareilly	216
UofLaval16	S333	Berta	223
UofLaval29	S256	Bovismorbificans	218
UofLaval12	S209	Braenderup	209
UofLaval33	S325	Bredeney	213
UofLaval14	S364	Cerro	200
UofLaval30	S1426	Cubana	222
UofLaval5	S229	Derby	218
UofLaval31	S718	Derby	223
UofLaval9	S354	Gaminara	198
UofLaval8	S219	Hadar	215
UofLaval24	S1603	Havana	208
UofLaval1	S1288	Javiana	206
UofLaval17	S551	Johannesburg	220
UofLaval3	S246	Kentucky	216
UofLaval19	S267	Kiambu	211
UofLaval25	S334	Kottbus	216
UofLaval6	S494	Lockleaze	192
UofLaval22	S238	Mbandaka	201
UofLaval13	S1395	Mississippi	207
UofLaval2	S1925	Muenchen	218
UofLaval15	S206	Muenchen	215
UofLaval27	S317	Ohio	203
UofLaval28	S307	Poona	197
UofLaval21	S212	Stanley	223
UofLaval18	S761	Stanleyville	218
UofLaval26	S193	Thompson	210
UofLaval34	S164	Typhimurium	226
UofLaval11	S277	Uganda	209

^a^ VG = Virulence gene.

**Table 6:** Relative distribution of core and variable virulence genes and their participation in the different stages of pathogenesis in *Salmonella enterica* (*n* = 69 strains).

Pathogenesis Stage	Virulence Genes		
	Core_69strains_	Variable_69strains_	Total
Attachment	25	24	49
Invasion	54	19	73
Intramacrophage survival	32	7	39
Systemic dissemination	44	22	66
Total	155	72	227

**Table 7:** Pathogenicity of 10 *Salmonella* Typhimurium strains in a chicken infection model and number of variant virulence genes detected with the AmpliSeq tool using Number 8 strain ST07-22495 as index.

ID/Group	Host	Antigens	Phage Typing	PFGE Patterns	Survival (7d)	Survival (>13d)	1st Sign	^b^ Variant Number
1: ST07-7666	Sparrow	4,5:12	160	XAI 0280/BNI 0071	50%	40%	3d	10
2: ST07-7670	Sparrow	4,5:12	160	XAI 0280/BNI 0071	90%	70%	3d	10
3: ST07-12345	Sparrow	4,5:12	160	XAI 0280/BNI 0071	100%	90%	3d	10
4: ST07-32529	Sparrow	4,5:12	160	XAI 0280/BNI 0296	60%	^a^ ND	2d	10
5: ST07-12335	Sparrow	4,5:12	146	XAI 0021/BNI 0096	70%	^a^ ND	2d	10
6: ST07-35522	Sparrow	4,5:12	40	XAI 0075/BNI 0297	60%	^a^ ND	2d	9
7: ST07-22792	Cormorant	4,5:12	1	XAI 0654/BNI 0295	100%	100%	13d	17
8: ST07-22495	Gull	4,5:12	41	XAI 0269/BNI 0081	60%	40%	2d	Index
9: ST07-24355	Gull	4,5:12	125	XAI 0269/BNI 0081	70%	40%	2d	1
10: ST07-29216	Chicken	4,5:12	126	XAI 0269/BNI 0081	100%	40%	3d	1

^a^ ND: not determined. ^b^ AmpliSeq tool results: variant number represents each instance of sequence variation among the virulence genes as detected following the mapping of the amplicon reads to the *Salmonella* Typhimurium reference genome.

## Data Availability

Not applicable.

## Supplementary Materials

The following supporting information can be downloaded at: www.mdpi.com/article/10.3390/microorganisms10020369/s1, Figure S1: Pulsed-field gel electrophoresis pattern of *Xba*I- and *Bln*I- digests of DNA fragments of 10 *Salmonella* Typhimurium strains that were used for pathogenicity assessment in a chicken infection model. Restriction fragments are shown following the digestion of DNA of each strain with *Xba*I (A) and *BIn*I (B), and electrophoresis on 1% agarose gel followed by staining for visualization. PFGE patterns (XAI No. and BNI No.) were designated using BioNumerics software after comparison to the PulseNet Canada database. Table S1: List of all 1200 primers used in the designed AmpliSeqSalm_227VG panel. Table S2: List of 16,729 amplicons including size, start/stop locations of in silico generated amplicons used for the designed AmpliSeqSalm_227VG panel. Table S3: List of variants of virulence genes detected among 29 out of 35 *Salmonella enterica* strains bioinformatically assigned to the correct serovar. Table S4: Presence and absence of the 227 virulence genes among all the tested 69 *Salmonella* strains. Table S5: Variants of the virulence genes found in 10 *Salmonella* Typhimurium strains used for an infection experiment based on No. 8_ST07-22495 as reference.
