# Supplementary material for: Application of a High-Throughput Targeted Sequence AmpliSeq Procedure to Assess the Presence and Variants of Virulence Genes in Salmonella
Source: Microorganisms. 2022 Feb 5;10(2):369. doi: 10.3390/microorganisms10020369 (PMC8879106; doi:10.3390/microorganisms10020369)
Supplement: Supplementary file 1 [file microorganisms-10-00369-s001.zip › Figure S1.pptx]

## Slide 1
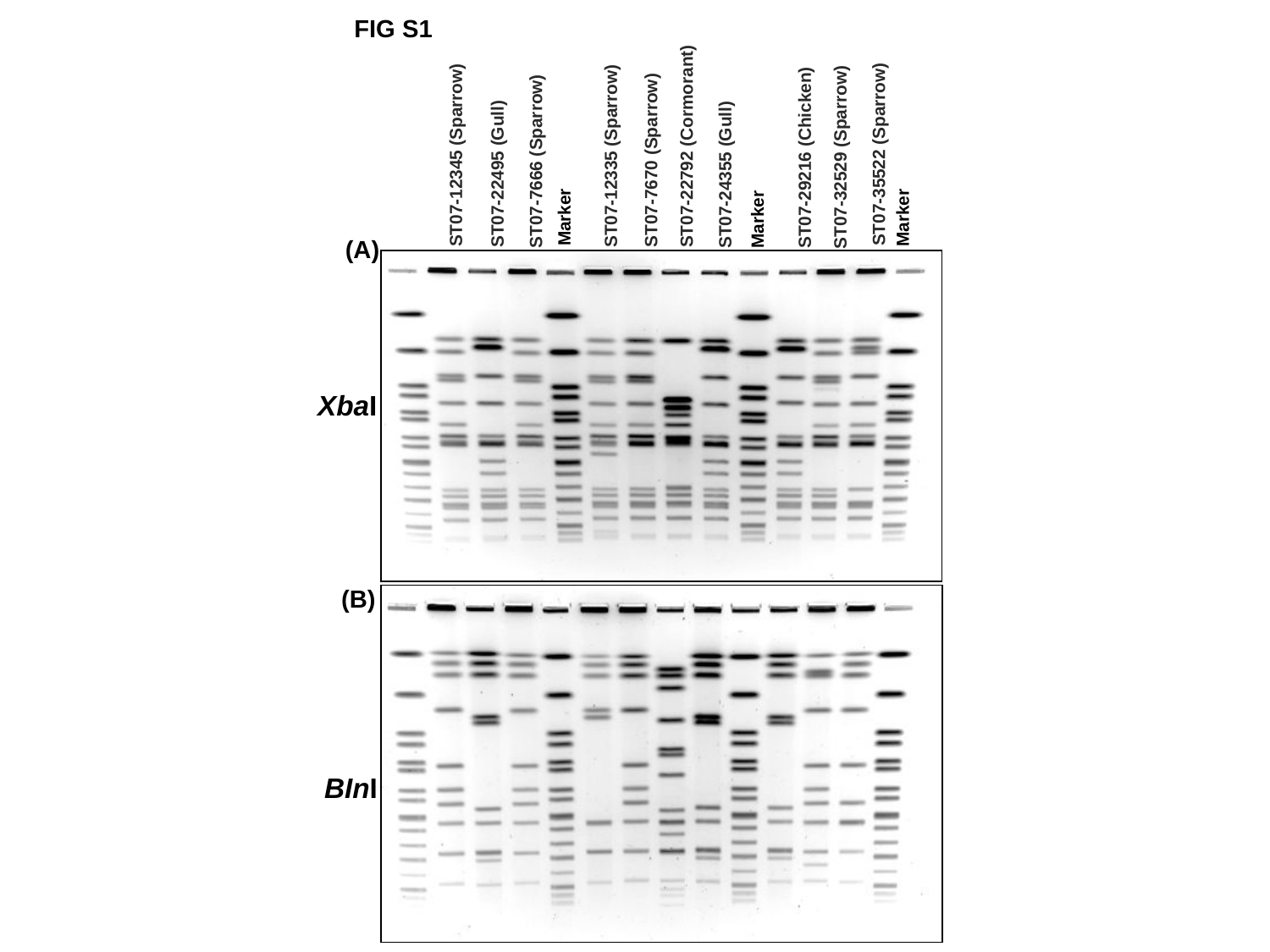

ST07-7670 (Sparrow)
ST07-22792 (Cormorant)
ST07-35522 (Sparrow)
ST07-12345 (Sparrow)
ST07-12335 (Sparrow)
ST07-22495 (Gull)
ST07-29216 (Chicken)
ST07-7666 (Sparrow)
ST07-32529 (Sparrow)
ST07-24355 (Gull)
Marker
Marker
Marker
(A)
XbaI
(B)
BInI
FIG S1
